# Making progress with the automation of systematic reviews: principles of the International Collaboration for the Automation of Systematic Reviews (ICASR)

**DOI:** 10.1186/s13643-018-0740-7

**Published:** 2018-05-19

**Authors:** Elaine Beller, Justin Clark, Guy Tsafnat, Clive Adams, Heinz Diehl, Hans Lund, Mourad Ouzzani, Kristina Thayer, James Thomas, Tari Turner, Jun Xia, Karen Robinson, Paul Glasziou, On behalf of the founding members of the ICASR group, Clive Adams, Olga Ahtirschi, Elaine Beller, Justin Clark, Robin Christensen, Heinz Diehl, Julian Elliott, Paul Glasziou, Sergio Graziosi, Joel Kuiper, Hans Lund, Rasmus Moustgaard, Annette O’Connor, Mourad Ouzzani, Jacob Riis, Karen Robinson, Karla Soares-Weiser, Kris Thayer, James Thomas, Tari Turner, Guy Tsafnat, Camilo Vergara, Ida Wedel-Heinen, Jun Xia

**Affiliations:** 1 Centre for Research in Evidence-Based Practice, Bond University, Robina, Australia; 2 Australian Institute of Health Innovation, Macquarie University, Sydney, Australia; 3 Faculty of Medicine and Health Sciences, University of Nottingham, Nottingham, UK; 4 Centre for Evidence-Based Practice, Bergen University College, Bergen, Norway; 5 Western Norway University of Applied Sciences, Bergen, Norway; 6 Qatar Computing Research Institute, Hamad Bin Khalifa University, Doha, Qatar; 7 National Institute of Environmental Health Sciences, PennState University, Pennsylvania, USA; 8 University College London, London, UK; 9 Monash University, Clayton, Australia; 10 JHU Evidence-based Practice Center, Johns Hopkins University, Baltimore, USA

**Keywords:** Systematic review, Automation, Collaboration

## Abstract

Systematic reviews (SR) are vital to health care, but have become complicated and time-consuming, due to the rapid expansion of evidence to be synthesised. Fortunately, many tasks of systematic reviews have the potential to be automated or may be assisted by automation. Recent advances in natural language processing, text mining and machine learning have produced new algorithms that can accurately mimic human endeavour in systematic review activity, faster and more cheaply. Automation tools need to be able to work together, to exchange data and results. Therefore, we initiated the International Collaboration for the Automation of Systematic Reviews (ICASR), to successfully put all the parts of automation of systematic review production together. The first meeting was held in Vienna in October 2015. We established a set of principles to enable tools to be developed and integrated into toolkits.

This paper sets out the principles devised at that meeting, which cover the need for improvement in efficiency of SR tasks, automation across the spectrum of SR tasks, continuous improvement, adherence to high quality standards, flexibility of use and combining components, the need for a collaboration and varied skills, the desire for open source, shared code and evaluation, and a requirement for replicability through rigorous and open evaluation.

Automation has a great potential to improve the speed of systematic reviews. Considerable work is already being done on many of the steps involved in a review. The ‘Vienna Principles’ set out in this paper aim to guide a more coordinated effort which will allow the integration of work by separate teams and build on the experience, code and evaluations done by the many teams working across the globe.

## Background

Systematic reviews (SR) are vital for both health practice and future research because they bring together all relevant evidence into one place, using transparent methods. However, such reviews have become ever more complicated, due to the complexity of interventions being studied and the amount of evidence being published which needs to be incorporated [1]. Systematic reviewers can no longer keep up with the ensuing workload using traditional manual methods of reviewing. With a median age of 8 months since last search, most systematic reviews are already outdated on publication [2]. The Cochrane Handbook for Systematic Reviews of Interventions recommends that the last search should be within 6 months of publication [3].

Fortunately, many of the tasks in a systematic review are amenable to automation: screening of titles and abstracts, sourcing full texts of included studies, data extraction and even collation of meta-analysis results are all fertile areas for automation [4]. Tools which streamline searching and citation screening could be used to quickly determine if new, eligible research has been carried out and should trigger an update of a SR. Recent advances in natural language processing, text mining and machine learning have produced new algorithms that can accurately mimic human endeavour in systematic review activity, faster and more cheaply [5–8].

However, development of automation tools from these algorithms has been slow and fragmented in large part because this type of work is difficult to fund. Not-for-profit research groups cannot afford to invest in the development of commercial products nor afford the ongoing licence fees of such products. Groups of researchers can sometimes find the funding to automate one or more small tasks in which they are experts or have a special interest in the basic science behind them (e.g. natural language processing, machine learning, library science). To fully reach the potential of automating systematic reviews, researchers will need a sustained coordinated collaborative effort, analogous to that of sequencing the human genome.

Automation tools need to be able to work together, to exchange data and results, so that systematic reviewers can choose the toolkit that best suits their review. Therefore, we initiated the International Collaboration for the Automation of Systematic Reviews (ICASR), to successfully put all the parts of automation of systematic review production together. The first meeting in Vienna in October 2015 was attended by information specialists, librarians, software engineers, statisticians, a linguist, artificial intelligence experts and researchers. We established a set of principles to enable tools to be developed and integrated into toolkits. This paper sets out the principles devised at that meeting.

## Methods

Prior to the meeting, a focussed literature search was conducted, in order to determine potential topics for the development of the principles. These were incorporated into a discussion document and circulated prior to the meeting.

The meeting was by invitation, based on the coordinators’ knowledge of automation tools and developers. Invitees could in turn invite others they knew to be involved in automation tool development. Twenty-four people attended. The meeting ran for a full day, adjacent to the Cochrane Colloquium. There was no sponsor.

Potential topics for principles were presented by the meeting coordinators, and preliminary discussion ensued. Small groups were formed by participants’ self-selection according to areas of expertise and knowledge. Within small groups, the topics were refined into the principles and draft wording written. At the end of the day, the wording from the small groups was presented and debated by the wider group, before being agreed upon.

Subsequent meetings of the ICASR group were held in Philadelphia in 2016 and London in 2017. These meetings focussed on the technical aspects of testing automation tools, the development of test data sets and the interoperability of different tools. Results from these discussions will be reported elsewhere. This paper outlines the principles the group has decided upon and the rationale underpinning them.

## The Vienna Principles

1. Systematic review production involves multiple tasks, each with different issues, but all must be improved.

**Fig. 1:**
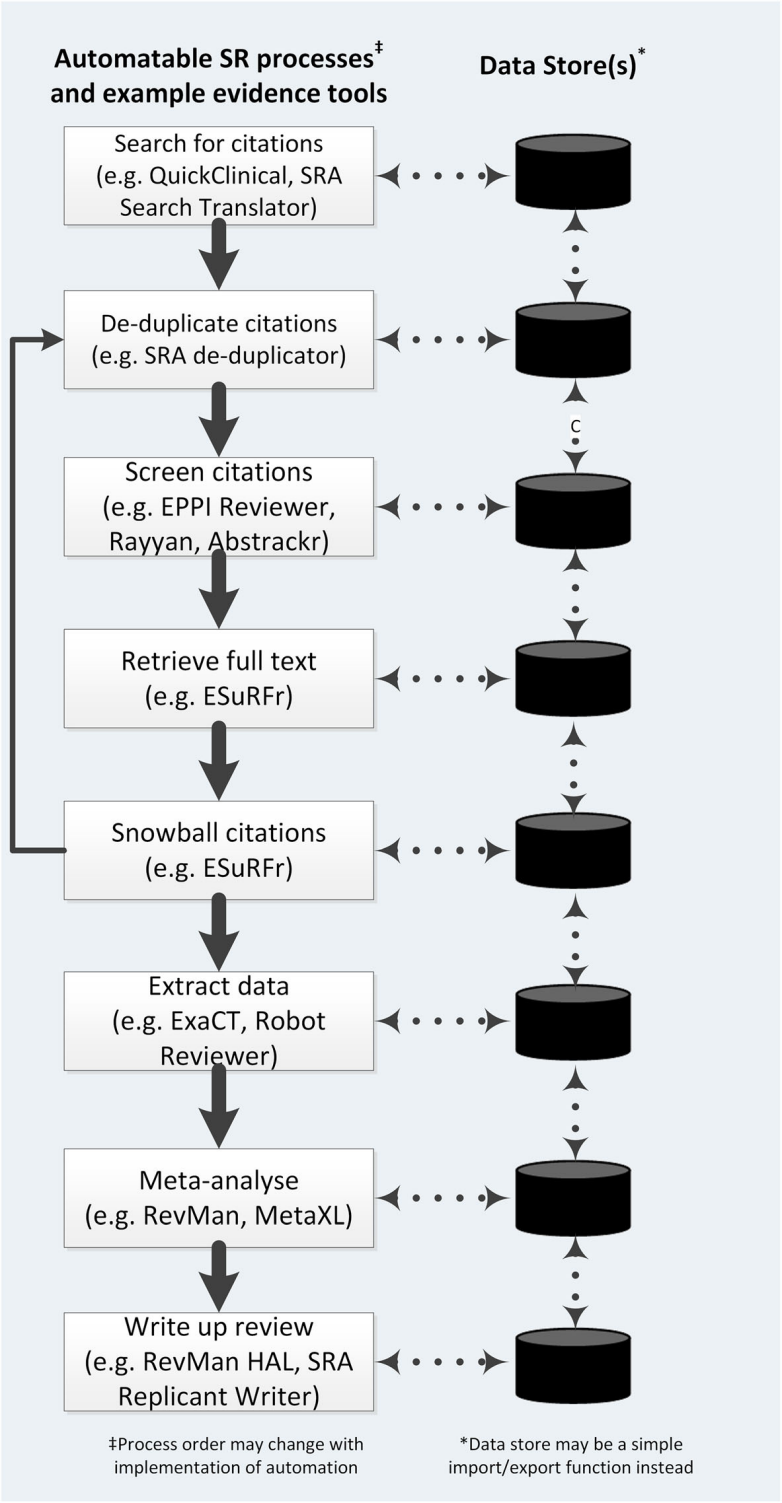
Automatable systematic review processes and example automation tools

There are four main tasks to be accomplished when conducting a SR which are amenable to automation: retrieving the relevant evidence, evaluating the studies, synthesising the evidence and publishing the review [4]. These four objectives have been broken down further into 15 distinct tasks [9]. To achieve the optimum speed and efficiency of producing SRs, automation technology needs to be used for each task amenable to automation (Fig. 1). Some tools under development were showcased at the meeting (Table 1). A more complete list of SR automation tools by ICASR members and others is maintained on a website [10]. To improve each task, we can use incremental and iterative software development techniques which have been proven successful in developing large software projects [11]. As automation can potentially help with many of the tasks in a review, we need to analyse each task and initially target those most suitable for efficiency improvements through automation. Automation of these tasks will allow more time for the important aspects of formulating an appropriate question, choosing suitable outcomes to study and interpreting results.

2. Automation may assist with all tasks, from scoping reviews to identifying research gaps as well protocol development to writing and dissemination of the review.

**Table 1:** Software tools showcased at the first meeting

Software or tool	Website or publication link	Task automated	Description
Covidence	https://www.covidence.org/	Screen citations	Initially designed for citation screening, it is now developing features like full-text reviewing, risk of bias assessment, extraction of study data and links directly with RevMan.
RevMan HAL	http://schizophrenia.cochrane.org/revman-hal-v4	Write up review	The writing of Cochrane reviews involves accurate copying of data from one part of a RevMan file to another. RevMan HAL has been designed to produce an automatic first draft of important sections of a Cochrane review. It uses already entered data from date of last search, analysis and summary of findings tables. to generate text for most sections of the abstract, summary of search, effects of interventions and summary of main results in the discussion section.
eSuRFr	http://www.ncbi.nlm.nih.gov/pmc/articles/PMC4211030/	Snowball citations	The proposed method for automatic citation snowballing is accurate and is capable of obtaining the full texts or abstracts for a substantial proportion of the scholarly citations in review articles. By automating the process of citation snowballing, it may be possible to reduce the time and effort of common evidence surveillance. tasks such as keeping trial registries up to date and conducting systematic reviews.
Rayyan	http://rayyan.qcri.org	Screen citations	The process begins with the author including or excluding a training set of studies. Then, an algorithm using computer learning starts to provide a ranking system for the remaining papers based upon the papers that have been selected.
EPPI-Reviewer	http://eppi.ioe.ac.uk/cms/Default.aspx?tabid=1913	Screen citations	EPPI-Reviewer acts as a reference manager, imports references in a wide variety of ‘tagged’ formats, conducts duplicate checking using ‘fuzzy logic’, stores documents, has direct searching of PubMed and has study classification and data extraction schemas with a multi-user interface to allow. comparison of results between researchers. It also contains ways of calculating common measures of effect (odds ratios, risk ratios, risk differences, standardised mean differences, mean differences) and can perform meta-analyses.
Evidence Pipeline	http://community.cochrane.org/tools/data-management-tools/evidence-pipeline	Screen full text	The Evidence Pipeline will address the difficulty in finding reports of studies for inclusion in a Cochrane review. The project will build an ‘Evidence Pipeline’ in which study citations identified through automated and enhanced centralised search activities, including Project Transform’s Getting. Involved platform, are ‘triaged’ to the most relevant review group or review using machine learning technologies.
Systematic Review Accelerator (SRA)	http://crebp-sra.com	Devise search strategy Search De-duplicate	A multi-function tool that currently enables researchers to analyse articles for building search strategies and translating search syntax between databases to speed up the search process. Also contains a de-duplicator to remove the need to manually de-duplicate the same studies from multiple databases.
Systematic Review Toolbox	http://systematicreviewtools.com	N/A	A comprehensive database of tools for automating and conducting systematic reviews is maintained by Dr. Chris Marshall, University of York.

A typical SR takes from 6 months to 2 years or more to write [12]. This extended production process has led to the proliferation of ‘rapid techniques’ such as writing rapid reviews [13] or conducting scoping studies [14] rather than conducting a SR which aims to identify the complete scope of the literature. It has been estimated that rapid reviews take around half the time of a typical SR [13]. Automation will mean that any review can be fully systematic and complete and performed in less time than rapid reviews take currently. However, it is important to not just focus on traditional SRs but to ensure the automation tools developed can be used in all aspects of evidence synthesis.

3. The processes for each task can and should be continuously improved, to be more efficient and more accurate.

The creation of automation tools for SRs is relatively recent. Hence, most tools are still in the early stages of development [15]. It is important to develop, evaluate and incrementally improve the techniques for all the tasks, with an initial focus on ‘bottlenecks’. The evaluations should involve both ‘laboratory’ and real-world testing, and hence, we should deploy tools as they are ready to allow user feedback drive their continual improvement.

4. Automation can and should facilitate the production of systematic reviews that adhere to high standards for the reporting, conduct and updating of rigorous reviews.

The point of developing and using automation tools and techniques is to create high quality SRs in a more timely fashion and with less effort. Furthermore, automation of processes means less involvement of people and has the potential to reduce human reviewer bias and mistakes during the preparation of a systematic review [16, 17]. In developing and testing new tools, it must be ensured that standards are kept high and new production approaches adhere to current SR guidelines such as the PRISMA Statement [18] and the Cochrane MECIR Standards for conduct and reporting of reviews [19].

5. Developments should also provide for flexibility in combining and using components, e.g. subdividing or merging tasks and allowing different users to use different interfaces.

Different types of SRs will have different needs. For example, a SR of qualitative studies is very different from a SR of randomised controlled trials, although many steps will be similar, such as title and abstract screening, and hence will potentially use common automation methods. Therefore, developing flexible tools will help use across different review types.

Automation tools should provide a clear output after each step of the process which will ensure that reviewers are not locked into using any single tool, but can use the sequence that best suits their purposes. Also, if the tools become unavailable or lose developer support, reviewers can move to alternatives for the specific tasks. This will require those building the tools to support commonly used import/export file types as well as allowing access via an API such as Representational State Transfer (ReST) which provides a well-documented standards framework to link various tools [20].

6. Different groups with different expertise are working on different parts of the problem; to improve reviews as a whole will require collaboration between these groups.

The automation of the SR process involves a variety of skills from a variety of specialists: information specialists, librarians, software engineers, statisticians, linguists, artificial intelligence experts and researchers from many different disciplines who are involved in the preparation of SRs. Combining the skills and knowledge of all these experts will improve the ability of automation techniques and tools, in much the same way that multidisciplinary teams in healthcare improve patient outcomes [21]. Teams in different organisations and contexts are working on similar elements of automation. There is much to be learned from the different approaches that are being taken; however, we should avoid unnecessary duplication of effort and leverage work from multiple groups, perspectives, and expertise. It is also important to be inclusive. For example, it may be an advantage that there are multiple tools for title and abstract screening as the varied writers of SRs may find one preferable over the other. ICASR is a step towards bringing the groups together.

7. Every automation technique should be shared, preferably by making code, evaluation data and corpora available for free.

While there may be use of modules developed by commercial groups, the collaboration should endeavour to make the tools publicly available and make the computing code of automation techniques open source so that others can re-use and build on previous work. Therefore, it is important to find public or philanthropic funding streams for the automation projects. Hence, an additional role of the collaboration is to provide support and guidance in securing these funding sources and developing a business model for sustainability. Successful collaboration may indeed bring about sustainable funding. In addition, the ability for groups working in this field to obtain academic credit for their work is important to motivation and for obtaining such funding. The benefits of sharing software and workflow techniques have been shown to reduce repetitive research tasks [22].

8. All automation techniques and tools should be evaluated using recommended and replicable methods and should report results and data.

Components of the automation process should be able to be independently evaluated by a third party. This means that the evaluation methods and corpora (the validated data sets from previous reviews) should be shareable and shared, free of charge. Not only technical specifications should be evaluated but also elements such as user interface and how well it fits into the SR workflow as a whole. This will prevent the potential bias that self-evaluation can bring [23] as well as provide outside users with confidence in the tools that are created [24]. ICASR is developing a repository of test data sets and results of testing. We aim to demonstrate thorough testing and replicability of results to all who use the automation tools we develop.

## Conclusions

Automation has a great potential to improve the speed of systematic reviews. Considerable work is already being done on many of the steps involved in a review. However, much of that work is done in isolation, resulting in software that cannot readily be integrated into a larger system, and is often neglected or abandoned. The ‘Vienna Principles’ set out in this paper aim to guide a more coordinated effort which will allow the integration of work by separate teams and build on the experience, code and evaluations done by the many teams working across the globe.

The development of the Vienna Principles highlighted several tensions and barriers to better coordination. For example, there is a tension between commercial and non-commercial development, between gaining appropriate individual academic credit and the common good and between different approaches to the overall problem. The principles attempt to address, but do not completely resolve, these tensions.

The principles suggested here do not address some of the technical aspects which still need solving. For example, there is often a need to be able to manually review and manipulate results from use of one automation tool before moving to the next tool. The use of multiple interlocking tools will demand a way of keeping an audit trail of tools used and changes made to data. As systematic reviews become more complex (e.g. network meta-analyses, complex interventions with multiple components) and searches become broader, the tools will need to handle large datasets. This may involve use of both web-based and stand-alone versions of tools or the use of large commercial data services.

Another area not yet addressed by these principles is the integration of systematic reviews into the knowledge translation process, such as the use of GRADE to assess evidence quality and the automated production of clinical practice guidelines.

Where to go next after the agreement on these principles? The Vienna meeting was the beginning of a more collaborative effort to share ideas, developments, tools and code. However, it will take time to develop trust, clear agreements, and collective action. A second meeting was held in Philadelphia in September 2016 and a third in London in 2017. In addition, ongoing community communication and infrastructure are needed to support discussions and exchanges. These are developing, but, as for much progress in science, such as the human genome project, the work will take a sustained collaborative effort.

## Abbreviations

API: Application Programming Interface
ICASR: International Collaboration for the Automation of Systematic Reviews
MECIR: Methodological Expectations for Cochrane Intervention Reviews
PRISMA: Preferred Reporting Items for Systematic Reviews and Meta-Analyses
ReST: Representational State Transfer
SR: Systematic review

## References

[1] Bastian H, Glasziou P, Chalmers I (2010). Seventy-five trials and eleven systematic reviews a day: how will we ever keep up?. PLoS Med.

[2] Beller EM, Chen JK, Wang UL, Glasziou PP (2013). Are systematic review up-to-date at the time of publication?. Syst Rev..

[3] Higgins JPT, Green S, editors. Cochrane Handbook for Systematic Reviews of Interventions Version 5.1.0 [updated March 2011]: The Cochrane Collaboration; 2011. Available from www.handbook.cochrane.org. Accessed 12 Dec 2017.

[4] Tsafnat G, Glasziou P, Choong MK, Dunn A, Galgani F, Coiera E (2014). Systematic review automation technologies. Syst Rev..

[5] Michelson JD, Pariseau JS, Paganelli WC (2014). Assessing surgical site infection risk factors using electronic medical records and text mining. Am J Infect Control.

[6] O'Mara-Eves A, Thomas J, McNaught J, Miwa M, Ananiadou S (2015). Using text mining for study identification in systematic reviews: a systematic review of current approaches. Systematic reviews.

[7] Marshall IJ, Kuiper J, Wallace BC (2016). RobotReviewer: evaluation of a system for automatically assessing bias in clinical trials. J Am Med Inform Assoc.

[8] Olofsson H, Brolund A, Hellberg C, Silverstein R, Stenstrom K, Osterberg M, Dagerhamn J (2017). Can abstract screening workload be reduced using text mining? User experiences of the tool Rayyan. Research Synthesis Methods.

[9] Tsafnat G, Dunn A, Glasziou P, Coiera E (2013). The automation of systematic reviews. BMJ.

[10] http://systematicreviewtools.com/index.php. Accessed 12 Dec 2017.

[11] Cockburn A (2008). Using both incremental and iterative development. STSC CrossTalk.

[12] Tsertsvadze A, Chen YF, Moher D, Sutcliffe P, McCarthy N (2015). How to conduct systematic reviews more expeditiously?. Syst Rev..

[13] Featherstone RM, Dryden DM, Foisy M, Guise JM, Mitchell MD, Paynter RA, Robinson KA, Umscheid CA, Hartling L (2015). Advancing knowledge of rapid reviews: an analysis of results, conclusions and recommendations from published review articles examining rapid reviews. Syst Rev.

[14] Arksey H, O'Malley L (2005). Scoping studies: towards a methodological framework. Int J Soc Res Methodol.

[15] Marshall C and Brereton P. Tools to support systematic literature reviews in software engineering: a mapping study. 2013 ACM/IEEE International Symposium on Empirical Software Engineering and Measurement, IEEE. 2013. https://ieeexplore.ieee.org/document/6681371/. Accessed 8 May 2018.

[16] Ho RS, Wu X, Yuan J, Liu S, Lai X, Wong SY, Chung VC (2015). Methodological quality of meta-analyses on treatments for chronic obstructive pulmonary disease: a cross-sectional study using the AMSTAR (Assessing the Methodological Quality of Systematic Reviews) tool. NPJ primary care respiratory medicine.

[17] Gomez-Garcia F, Ruano J, Gay-Mimbrera J, Aguilar-Luque M, Sanz-Cabanillas JL, Alcalde-Mellado P, Maestre-Lopez B, Carmona-Fernandez PJ, Gonzalez-Padilla M, Garcia-Nieto AV, et al. Most systematic reviews of high methodological quality on psoriasis interventions are classified as high risk of bias using ROBIS tool. J Clin Epidemiol. 2017;92:79–88. 10.1016/j.jclinepi.2017.08.015 28893571

[18] Moher D, Liberati A, Tetzlaff J, Altman DG, PRISMA Group (2009). Preferred reporting items for systematic reviews and meta-analyses: the PRISMA statement. PLoS Med.

[19] Higgins J, Lasserson T, Chandler J, Tovey D, Churchill R. Standards for the conduct and reporting of new Cochrane Intervention Reviews, reporting of protocols, and the planning, conduct and reporting of updates. 2016. http://community.cochrane.org/mecir-manual. Accessed 18 Apr 2017.

[20] Fielding RT. Architectural styles and the design of network-based software architectures (dissertation). University of California, Irvine. https://www.ics.uci.edu/~fielding/pubs/dissertation/top.htm. Accessed 18 Apr 2017.

[21] Epstein NE (2014). Multidisciplinary in-hospital teams improve patient outcomes: a review. Surg Neurol Int.

[22] De Roure D, Goble C (2009). Software design for empowering scientists. IEEE Softw.

[23] Althubaiti A (2016). Information bias in health research: definition, pitfalls, and adjustment methods. J Multidiscip Healthc.

[24] Rathbone J, Carter M, Hoffmann T, Glasziou P (2015). Better duplicate detection for systematic reviewers: evaluation of Systematic Review Assistant-Deduplication Module. Systematic reviews.

